# Epistemic injustice in psychiatry

**DOI:** 10.1192/pb.bp.115.050682

**Published:** 2017-04

**Authors:** Paul Crichton, Havi Carel, Ian James Kidd

**Affiliations:** 1 Ministry of Justice, London, UK; 2 University of Bristol; 3 Nottingham University

## Abstract

It has been argued that those who suffer from medical conditions are more vulnerable to epistemic injustice (a harm done to a person in their capacity as an epistemic subject) than healthy people. This editorial claims that people with mental disorders are even more vulnerable to epistemic injustice than those with somatic illnesses. Two kinds of contributory factors are outlined, global and specific. Some suggestions are made to counteract the effects of these factors, for instance, we suggest that physicians should participate in groups where the subjective experience of patients is explored, and learn to become more aware of their own unconscious prejudices towards psychiatric patients.

Epistemic injustice is a harm done to a person in her capacity as an epistemic subject (a knower, a reasoner, a questioner) by undermining her capacity to engage in epistemic practices such as giving knowledge to others (testifying) or making sense of one's experiences (interpreting). It typically arises when a hearer does not take the statements of a speaker as seriously as they deserve to be taken. The prime case of epistemic injustice is testimonial: the hearer deflates the level of credibility she gives the speaker because she is (often unconsciously) prejudiced against the social group to which the speaker belongs. Common examples include sexism and racism. In such cases the testimony of a woman or a person from an ethnic minority background will be given deflated credibility, based on the prejudicial associations between that group and negative stereotypes. The reason we chose this approach is that epistemic injustice provides an account for why, despite the best intentions, physicians often do not believe what psychiatric patients tell them. Clarifying some of the reasons for this common prejudicial stereotype of patient unreliability may make it possible to explore ways of overcoming the epistemic injustice that we suggest patients, and in particular psychiatric patients, are vulnerable to.

It is worth noting here that it is prejudicial or negative stereotypes, not stereotypes *per se*, which often give rise to epistemic injustice. We rely on stereotypes as heuristic aids in making credibility judgements because they are often empirically reliable generalisations. However, negative attitudes towards people with a mental illness may lead to negative stereotypes and to generalisations which are resistant to counter-evidence, owing to what philosopher Miranda Fricker calls an ‘ethically bad affective investment’.^1^ It is these kinds of stereotypes that may lead to epistemic injustice.

## Epistemic injustice and psychiatric patients

Epistemic injustice is important in psychiatry because of the persistent negative stereotypes that affect people with mental disorders in particular and lead to a credibility deficit. The consequence is that patient testimonies and interpretations are not acknowledged as credible, and patients are thus undermined in their capacity as knowers and contributors to the epistemic effort to reach a correct diagnosis and treatment. We suggest that people with mental disorders are even more susceptible to epistemic injustice than those with physical illnesses, for reasons that are detailed below.

We have argued in the past that people with physical illnesses are vulnerable to epistemic injustice.^2,3^ Here we suggest that people with mental disorder may be susceptible to even greater epistemic injustice than people with physical illnesses. This is mainly owing to the high prevalence and great power of negative stereotypes of psychiatric illness. As a consequence, the patient may be telling the truth, but the doctor deflates the level of credibility which she gives to the patient (‘credibility deficit’) and thereby does the patient a distinctive kind of injustice, namely epistemic injustice, which undermines the patient specifically in her capacity as a giver of knowledge. This has detrimental effects on individual psychiatric patients, but also on the funding of psychiatric services and the public perception of mental disorder. Many people are influenced by negative stereotypes about mental disorders, are ill informed about their true nature, and have little understanding of how to treat them. Some measures to improve the current situation are suggested below.

### Measures to counter epistemic injustice

The notion of epistemic injustice has been developed by Fricker.^1^ She was interested in how social identity and power affects people's status as knowers. She gives the example of a White police officer who stops a Black car driver and asks him whether the car belongs to him. If the driver truthfully says that the car is his, but the policeman does not believe him because of racial prejudice, then he does the driver an injustice in his capacity as a knower.^4^

The main type of epistemic injustice that Fricker discusses is ‘testimonial injustice’; it emerges from the fact that testifying, i.e. giving information to others, depends crucially on one's perceived credibility. If a person is seen as lacking credibility, her testimony will be ignored or treated with suspicion, or it might not be solicited at all. Of course, she may lack credibility for a good reason, for instance if she is a known liar. However, testimonial injustice occurs when a person suffers a ‘credibility deficit’ owing to some negative stereotype or prejudice associated with her social group (e.g. gender or race). This credibility deficit is unjustified and hence constitutes an epistemic injustice. Fricker analyses how negative racial and sexist prejudices unfairly deflate the credibility of people of Black and minority ethnic background and women, such that what they say is ignored, marginalised or otherwise excluded from epistemic consideration. Since being able to give information to others is essential to social life and agential action, testimonial injustice harms those who experience it.

Carel and Kidd have argued that people with physical illnesses are more vulnerable to epistemic injustice than healthy people.^2,3^ The testimonies of patients are often presumed to be irrelevant, unreliable, confused or otherwise lacking in credibility, owing to negative stereotypes associated with ill persons. Such stereotypes include viewing ill persons as cognitively impaired or emotionally compromised, owing either to their somatic condition or to their psychological reactions to it; or as existentially unstable, gripped by anxieties such that they ‘cannot think straight’; or as psychologically dominated by their illness in a way that warps their capacity to accurately describe and report their experiences (e.g. ‘the moaner’ or ‘the drama queen’ stereotype). Because illness often evokes strong feelings in those affected, their emotions are often taken by health professionals to have a detrimental effect on patients' thinking, distorting the accounts they give of their illness. This pattern may be more acute in cases where subjective symptoms are driving the clinical encounter, such as unexplained breathlessness (see www.lifeofbreath.org), chronic pain, or other medically unexplained symptoms.^5^

Of course, the credibility of an individual is context dependent: if someone is talking about a subject on which she is an acknowledged expert then she is much more likely to be believed than if she is talking about something she is known to have little knowledge of.

### Epistemic injustice – real-life situations

We are sufficiently aware of the existence of people's unconscious desires and beliefs to know that they can be mistaken about their own desires and beliefs, but it is also the case that they have exclusive access to many of their desires and beliefs. In the interests of epistemic justice, physicians should accept what people with mental disorders say about these matters as true unless there is good reason not to. Moreover, psychiatric patients who have experience of psychiatric services become reluctant to disclose psychotic symptoms because they know it might make them more likely to be diagnosed with a psychotic illness, and in some cases detained in hospital and medicated against their will. If they nonetheless disclose such symptoms, then psychiatrists might conclude that the symptoms are more severe in the sense that the patients are unable to inhibit their expression and/or that their executive function is also impaired.

Here we give three examples of epistemic injustice affecting psychiatric patients (Boxes 1, 2 and 3). Their purpose is to show that epistemic injustice can be a real problem in psychiatry, with possibly devastating effects on the individuals who are telling the truth. The personal details of the patients concerned have been altered to preserve their anonymity.

One of the important factors which can predispose to epistemic injustice is a widespread misunderstanding of the relationship between emotion and cognition, and the positive contribution made by emotional input to a broader conception of rationality.^6^ A consequence, in a medical and psychiatric context, is that the ‘soft evidence’ offered by patients is often met by credibility deflation. In practice this may lead to patient reports being ignored or discounted, especially when time pressure and other constraints are at play. Conversely, if the ‘hard evidence’ provided by objective investigation (e.g. blood tests) is regarded as more reliable, then the opinions of health professionals who can access and interpret that evidence may enjoy credibility inflation. In some cases it may be better for the doctor to try to treat on the basis of the symptoms reported by the patient rather than on the basis of an abnormal blood test result or an abnormal scan alone. An example of this is the PSA (prostate-specific antigen) test, which is a notoriously unreliable guide for the treatment of prostate cancer.

**Box 1** Example of epistemic injustice in psychiatry 1

When one of the authors (P.C.) was a medical student in Munich, Germany, he saw a young man on an acute psychiatric ward who said he was a relative of the then Soviet leader. The responsible consultant took this to be a grandiose delusion, and therefore as evidence of a psychotic illness; it later turned out to be true.

**Box 2** Example of epistemic injustice in psychiatry 2

The second example is of a woman in her early 50s, a former nun. The police contacted mental health services because they had been alerted by someone doing work on her house. They found evidence of smoke damage to the house, but not of fire damage. She was admitted to a psychiatric ward and detained under section 2 of the Mental Health Act. She claimed that she had been burning incense in the house for many years to drive away evil spirits. During the week she had spent on the ward there was no evidence of her trying to ward off evil spirits or attempting to start a fire, or of any psychotic symptoms. The mental health tribunal members concluded that her beliefs about incense and evil spirits were compatible with her religious faith, that there was no evidence of a psychotic illness, as had been claimed by the psychiatrist and one of the psychiatric nurses, and that section 2 should therefore be rescinded.

**Box 3** Example of epistemic injustice in psychiatry 3

The third example is of a young man who was admitted to psychiatric hospital on section 2 despite the fact that he had agreed all along to be admitted and remain in hospital as a voluntary patient. He had been standing near the edge of a high cliff for about an hour until passers-by called the police. The staff involved in his care on admission did not believe that he could be trusted to remain in hospital on a voluntary basis and argued in the tribunal for the maintenance of the section. His community psychiatric nurse attended the tribunal, stating that he should never have been placed on a section, because he had had suicidal thoughts for many years, had gone to the same cliff many times in the past, had been admitted to hospital on several occasions as a voluntary patient, and had misgivings about the stigma attached to being placed on a section. All this had been documented in the hospital notes. She conceded that there would always be a risk of self-harm, but that it was a matter of managing the risk without compulsory detention and with the help of his friends and family. After hearing this evidence the tribunal members decided to rescind the section.

A psychiatric example is to do with making a diagnosis of epilepsy. Here a patient may have some epileptiform waves on the electroencephalogram (EEG), but unless there is also clinical evidence of altered consciousness and/or involuntary movements which fit into a recognised pattern, a diagnosis of epilepsy cannot be made. An EEG can confirm but cannot exclude the diagnosis, which is essentially clinical.^7^

In very general terms, there are two types of contributory conditions for epistemic injustice affecting people with mental disorders: global and specific. Global factors are those that can affect any patient at risk of psychiatric disorder or those diagnosed as having psychiatric disorders. The fear of stigma among those at risk can make early intervention difficult and those who have been diagnosed may avoid service use and relapse more frequently.

## Global contributory conditions for epistemic injustice

There are three global contributory conditions for epistemic injustice in psychiatric illness:

1. problems associated with, and partly caused by, the mental disorder
2. the higher value placed by health professionals on ‘hard’ or objective evidence compared with patient reports
3. the entrenched negative stereotypes associated with mental disorders.

### 1. Problems related to mental disorder

Psychiatric patients are often disadvantaged – cognitively, socially and economically – and these disadvantages are frequently thought to be the patient's fault. People with mental disorders are often badly educated because the illness has interrupted their education (‘dropouts’); they are often financially impoverished because the effects of the illness may make them unemployable (‘lazy’, ‘dependence culture’); and they are frequently socially isolated (‘loners’). They may become dependent on substances such as nicotine, alcohol and street drugs (‘lack of willpower’) and frequently experience physical illnesses. Causes of physical illnesses include substance misuse, self-neglect secondary to mental disorder and/or substance misuse, and psychotropic medication, such as atypical antipsychotics causing cardiovascular problems (‘down to lifestyle’).

People with mental disorders are thus often seen to have largely brought these disadvantages on themselves and are stigmatised and held responsible for them,^1^ even though some conditions contributing to mental disorders, such as genetic factors and a dysfunctional environment, are outside the person's control. To the extent that such negative stereotypes are shared by their voters, politicians, who often look to save public money, will not be motivated to redress the imbalance in mental health funding: in 2010/2011 mental health services were allocated only 10.8% of the National Health Service (NHS) budget, although mental disorders constituted nearly 22.8% of the disease burden in the NHS.^9^ Simon Wessely, the president of the Royal College of Psychiatrists, notes that despite rising demand, spending on adult mental health by NHS trusts has fallen by 8% since 2010.^9^ A recent parliamentary report advocates ‘whole person’ care, which includes mental and physical health, and highlights some of the barriers to parity of esteem for mental health.^10^

### 2. Hard *v.* soft evidence

Health professionals are trained to place higher value on ‘hard’ or objective evidence, namely the results of investigations, than on ‘soft’ or subjective evidence provided by patients. In fact, some such objective evidence (e.g. from X-rays or magnetic resonance imaging (MRI) scans) is heavily dependent on interpretation, for instance by a radiologist. This gives health professionals epistemic power, because only they have access to this evidence and have the training to interpret it. Montgomery^11^ has argued that medicine is not itself a science but rather an interpretive practice that relies on clinical reasoning. A physician looks at the patient's history along with the presenting physical signs and symptoms and juxtaposes these with clinical experience and empirical studies to construct a tentative account of the illness with what Montgomery calls ‘clinical judgment’. In psychiatry, there is virtually no hard evidence and diagnoses have to be made mainly on the basis of what patients say and how they behave. However, some psychiatrists regard their patients as objects of their epistemic enquiry rather than participants in an epistemic search for the correct diagnosis and best treatment. Anthropologist Tanya Luhrmann^12^ argues that insurance companies exercise a more powerful influence over the content of healthcare than do doctors, in that they promote a biological approach to psychiatry because it yields explicit therapeutic rationales, targeted treatments and quantifiable outcomes that can be audited more easily.

Despite the lack of objective evidence in psychiatry, many psychiatrists are influenced by their general medical training and import this bias into the field. Although many acknowledge the biopsychosocial model of mental disorders, they often retain their biological orientation.^13^ Biological psychiatry has been dominant since the 1950s, when the first antipsychotic drugs were introduced, and there is little evidence that this is changing in any significant way. This is partly because the biological approach has practical benefits (e.g. psychiatrists can save time by focusing on drug treatments). Based on his experience working as a liaison psychiatrist in a large medical hospital, one of the authors (P.C.) believes that psychiatry itself is stigmatised within medicine and that some psychiatrists feel that they will be more respected by their medical colleagues if they approach mental disorders from a biological perspective. P.C. also senses that some patients might prefer this attitude, feeling exonerated if they are told that their mental disorder is caused by a ‘chemical imbalance in the brain’ which can be ameliorated by a drug.

### 3. Negative stereotypes

People with mental disorders are socially stigmatised and are frequently described with derogatory terms such as ‘mad’, ‘crazy’ or ‘weird’. The term ‘stigma’ comes from the ancient Greek word denoting the mark made on slaves by a pointed instrument. Stigma involves negative associations that attach to a social group. Sociologist Erving Goffman^14^ argued that stigmatised people are considered abnormal by society and are not fully socially accepted. As a consequence, they constantly try to adjust their social identities. These additional cognitive and social burdens increase the pressures on stigmatised people, exacerbating their already difficult social and cognitive situation. Thornicroft^15^ points out that patients often describe the stigma they encounter as worse than the mental disorder itself. Stigma affects every aspect of their lives, including employment, accommodation, financial resources and sense of citizenship. It is a major problem throughout the world.

One of the negative stereotypes associated with mental illness is that people with a mental illness are responsible for their condition. For example, people diagnosed with depression are often told to ‘get a grip’ or to ‘pull themselves together’. Illness, not only mental illness, is often seen as a mark of moral, social and epistemic failure (e.g. drug/alcohol dependence is sometimes seen as weakness of will). Such failures are shaped by group-specific values and commitments – for instance, certain religious groups regard depression as a punishment by God for their sins.^16^

However, in a legal setting, the poor insight of patients into their mental state may be recognised by the court as a factor which reduces the patient's responsibility for their actions. Although this diminishes their epistemic status, it also protects them, so recognition of their diminished responsibility may lead to them being treated in hospital rather than imprisoned.

Thus, those who are influenced by negative stereotypes about psychiatric patients may feel justified in cutting funding for mental health services because they think that many psychiatric patients are to blame for their mental health and other problems. In the case of depression, many people who have no personal experience of the illness tend to think that depressed people only need to think more positively for their depression to disappear.^17,18^ The fact that psychiatric services are more poorly funded than other services in the NHS suggests that negative stereotypes about mental disorders may have a role in funding distribution. These negative stereotypes are also influential in the broader context of widespread ignorance about the true nature of mental disorders and their treatment.

### Types of stigma and their effects

#### General stigma

General stigma has negative effects on the prevention, early intervention and treatment of mental disorders. The formulation of a diagnosis has the advantage of making resources available for treatment, as well as providing the best available treatment. Moreover, there is evidence that early treatment improves the prognosis (e.g. in schizophrenia).^7^ On the other hand, having a diagnosis also leads to stigma and discrimination, which can act as a barrier to recovery, for instance making it more difficult to find employment and accommodation.^15^

#### Self-stigma

People with mental illness often accept and internalise negative stereotypes, and this in turn leads to low self-esteem, shame, demoralisation, confidence loss and giving up goals.

#### Structural stigma and discrimination

Patients typically report that they feel their views are not sufficiently elicited or considered by those who plan and organise psychiatric services.^19^ We have already seen that psychiatric provision for approaches other than the biopsychosocial model is severely under-resourced.

## Specific contributory conditions

So far we have discussed global contributory conditions for epistemic injustice. In addition to these global conditions, there are specific problems which can lead to further kinds of epistemic injustice as a consequence of the particular nature of the mental disorder in question. Here are two examples, which illustrate how the symptoms of particular disorders may reduce the credibility of what patients report about their own experiences to an extent that constitutes epistemic injustice.

### Dementia

The first example is dementia, an acquired impairment of cognitive function without impairment of consciousness. The central feature of its commonest form, Alzheimer's disease, is memory loss, especially of episodic memory, but there can be a wide range of other cognitive impairments as well. The main negative stereotype associated with dementia is the belief that the impairment of cognitive function is severe and global; that the person has or will rapidly and inevitably become a ‘vegetable’. In fact, this is hardly ever the case, except perhaps in the final stage of the illness.^20^

The personality of the individual and some cognitive functions are often well preserved. Thus, patients with mild to moderate dementia can be much more reliable informants than they are often thought to be. There is a need for careful neuropsychological assessment to establish the severity of the impairment. If a person's memory is badly affected, much can be gained by staying in the present in conversations, thereby minimising the occurrence of behavioural markers of epistemic incapacity that can exacerbate the risk of epistemic injustice.^20^

### Schizophrenia

The second example is schizophrenia. Perhaps the most common stereotype associated with it is that because of their delusional beliefs, people with schizophrenia are unpredictable and violent. This may diminish their status as truth-tellers because it may be concluded from one false (delusional) belief that none of their beliefs are credible. In fact, although violent behaviour can occur in schizophrenia, it is much rarer than is thought. There is a small but significant increase in violence in patients with schizophrenia (in any one year 8% of such patients will commit an act of violence compared with 2% of the general population). There is, however, a much stronger association between violence and substance misuse than with schizophrenia. The proportion of all violent acts committed by those with schizophrenia is 3–4%. This leaves 96–97% of all violent acts committed by people who do not have this disorder. The risk of an individual patient with schizophrenia committing homicide is less than 1 in 3000. Moreover, the rates of suicide are much higher than homicide rates in psychiatric patients as a whole.^21^ Thus, although the risk of violence is much higher in patients with schizophrenia than in the general population, the risk is lower than is suggested in the media.^21^ It also seems likely that other factors apart from the illness itself may play a part, such as the influence of alcohol and illicit substances at the time of the offence, and social factors.

Such negative stereotypes are problematic for several reasons, beyond their empirical inadequacy. They encourage unwarranted attitudes of suspicion and distrust towards people with schizophrenia, which, in turn, can contribute to their social isolation; this is in itself epistemically impairing. Many of our epistemic practices are intrinsically social, such as testifying (giving information to others) and interpreting (making sense of one's experiences), and it is no coincidence that Fricker focuses her analysis of epistemic injustice on those two practices.^1^ Social isolation and epistemic impairment can be mutually reinforcing.

In the case of schizophrenia, this problem takes on a specific form: it is integral to our social and epistemic agency that other people perceive us as a person – an agent – capable of engaging, in a sustained and reasonable way, in testifying, interpreting and other epistemic practices. A self is a locus of epistemic and social agency. Yet stereotypes about schizophrenia abide, typically the widespread but mistaken notion that schizophrenia is chiefly characterised by a personality split, as in the good Dr Jekyll and the evil Mr Hyde. The term ‘schizophrenia’ was coined by the psychiatrist Eugen Bleuler to capture a split between components of the mind – knowledge, emotion and will. This idea of a split has been abandoned in modern diagnostic criteria.^22^ However, the stereotype of ‘split personality’ is, of course, a perfect example of a fragmented epistemic self with whom one cannot effectively engage either socially or epistemically.

The rare cases of homicide by patients with schizophrenia are given intense coverage in the press. Examples include Christopher Clunis, who killed a stranger who happened to be standing on the same platform at Finsbury Park tube station in London in 1992; and Matthew Williams, who had a diagnosis of paranoid schizophrenia and killed a young woman in an act of cannibalism in 2014. This creates the impression that violence on the part of patients with schizophrenia is much more common than in fact it is, a phenomenon described by psychologist Daniel Kahneman as ‘what you see is all there is’, namely jumping to conclusions from limited evidence: another feature of prejudice that might lead to epistemic injustice.^23^ Such jumping to conclusions on limited evidence can lead to prejudice (‘people with schizophrenia are violent’) and hence to epistemic injustice, if a patient says she does not have violent thoughts and is not believed.

As demonstrated in this section, the specific deficits found in dementia and schizophrenia can increase the susceptibility of such patients to epistemic injustice, in addition to the global factors which apply to all mental disorders.

## Possible ways of overcoming epistemic injustice

One effective way to integrate the subjective perspective of patients into medicine and psychiatry may be changes in medical and psychiatric training with a view to emphasising the psychological aspects of patient care. ‘Schwartz rounds’, which allow health professionals to focus on the existential, ethical and personal aspects of a medical case, are growing in popularity in the UK. We suggest that this approach should not only be taught to medical students but should become part of clinical practice.^24^ Regular interpersonal dynamic meetings with members of a multidisciplinary team, which create a forum for discussing problematic emotional contacts with patients, can enhance understanding of these aspects of patient care and reinforce their importance.^13^

Medical students should be taught to believe what psychiatric patients tell them, unless there is good reason not to do so. Students are frequently told to put patients first, but the experience of many patients is that they are often treated as cases rather than people, and that what is important to doctors is different to what is important to patients. By listening carefully to what patients tell them, doctors can make a conscious effort to imagine how things seem from the patient's perspective. In this way the relationship can become a genuinely collaborative one, rather than one in which the doctor decides what is in the patient's best interests.^25^

Fricker^1^ notes that hearers, in this case the physicians, need to practise giving more credibility to members of groups they fear they may be giving too low levels of credibility to: in this context, to psychiatric patients. Hearers may become aware of a cognitive dissonance: they may notice that on occasions they fail to live up to their belief that members of these groups are to be taken seriously, and then make a conscious effort to give them a higher level of credibility. The hope is that, with time, this corrective policy will become second nature.

## Conclusions

We have suggested that there is even greater risk of epistemic injustice in psychiatry than in general medicine. There is a need for psychiatrists to be trained to listen carefully to what patients are telling them and to engage with them in collaborative decision-making, to allow patients to have a greater epistemic role and to overcome the risk of epistemic injustice. Changes are also required in the social and political arena. Media editors should reduce the stigmatisation of psychiatric patients in media reports, especially if epistemic failure (such as reliance on negative stereotypes) can be a cause of moral failure (such as treating persons with mental disorders in an unfairly hostile or suspicious manner). Similarly, politicians should ensure that there is a fairer distribution of healthcare resources, not merely to mitigate the economic cost of mental ill health.

Prejudices against people with mental disorders are entrenched in our society in what Fricker calls the ‘collective social imagination’.^1^ They go unchecked because they operate below the radar of the conscious scrutiny of our own beliefs. Those who are in a position to influence public opinion have a special responsibility to oppose these prejudices. We hope that this editorial will increase awareness of the risks of epistemic injustice in psychiatry and thus contribute to this goal.

## References

[1] Fricker M Epistemic Injustice: Power and The Ethics of Knowing. Oxford University Press, 2007.

[2] Carel H Kidd IJ Epistemic injustice in healthcare: a philosophical analysis. Med Health Care Philos 2014; 17: 529–40. 24740808 10.1007/s11019-014-9560-2

[3] Kidd IJ Carel H Epistemic injustice and illness. J Appl Philos 2016; 34: 172–90. 28303075 10.1111/japp.12172 PMC5324700

[4] Fricker M Miranda Fricker on credibility and discrimination. In Philosophy Bites (eds. Edmonds D Warburton N). Oxford University Press, 2010.

[5] Carel H Macnaughton J Dodd J The invisibility of breathlessness. Lancet Respir Med 2015; 3: 278–9. 25890648 10.1016/S2213-2600(15)00115-0

[6] Crary A Beyond Moral Judgment. Harvard University Press, 2007.

[7] Cowen P Harrison P Burns T Shorter Oxford Textbook of Psychiatry: p. 6 Oxford University Press, 2012.

[8] Royal College of Psychiatrists Achieving Parity of Esteem between Mental and Physical Health. RCPsych, 2012 (http://www.rcpsych.ac.uk/pdf/Parity%20of%20Esteem%20briefing%20Feb%202012.pdf).

[9] Cooper C Britain's top psychiatrist Simon Wessely challenges Government to ring-fence mental health spending. Independent, 2 4 2016.

[10] Millard C Border P *Parity of Esteem for Mental Health* (POSTnote POST-PN-485). Parliamentary Office of Science and Technology, 2015.

[11] Montgomery K How Doctors Think: Clinical Judgment and the Practice of Medicine. Oxford University Press, 2005.

[12] Luhrmann TM Of Two Minds: The Growing Disorder in American Psychiatry. Alfred A. Knopf, 2000.

[13] Yakeley J Hale R Johnston J Kirtchuk G Schoenberg P Psychiatry, subjectivity and emotion – deepening the medical model. Psychiatr Bull 2014; 38: 97–101. 10.1192/pb.bp.113.045260 PMC4115376 25237517

[14] Goffman E Stigma: Notes on the Management of Spoiled Identity. Simon and Schuster, 1963.

[15] Thornicroft G Shunned: Discrimination against People with Mental Illness. Oxford University Press, 2006.

[16] Scrutton AP Two Christian theologies of depression (with peer review commentaries by Ian Kidd and John Swinton). Philos Psychiatr Psychol (in press).

[17] Smith B Depression and motivation. Phenom Cogn Sci 2013; 12: 615–35.

[18] Ratcliffe M What is it to lose hope? Phenom Cogn Sci 2013; 12: 597–614.

[19] Rüsch N Thornicroft G Does stigma impair prevention of mental disorders? Br J Psychiatry 2014; 204: 249–51. 24692749 10.1192/bjp.bp.113.131961

[20] Clarke C Howard R Rossor M Shorvon S (eds). Neurology: A Queen Square Textbook. Wiley-Blackwell, 2009.

[21] Frith C Johnstone E Schizophrenia: A Very Short Introduction. Oxford University Press, 2003.

[22] Crichton P ‘A profound duplicity of life’: uses and misuses of ‘schizophrenia’ in popular culture and professional diagnosis. Times Lit Suppl 2000; 21 3.

[23] Kahneman D Thinking, Fast and Slow. Penguin Books, 2011.

[24] The King's Fund Schwartz Center Rounds. Available at http://www.kingsfund.org.uk/projects/schwartz-center-rounds (accessed 9 June 2016).

[25] Smith R Thoughts for new medical students at a new medical school. BMJ 2003; 327: 1430–3. 14684637 10.1136/bmj.327.7429.1430 PMC300793

